# Understanding vaccination communication between health workers and parents: a Tailoring Immunization Programmes (TIP) qualitative study in Serbia

**DOI:** 10.1080/21645515.2021.1913962

**Published:** 2021-05-25

**Authors:** Vesna Trifunović, Katrine Bach Habersaat, Darija Kisić Tepavčević, Verica Jovanović, Milena Kanazir, Goranka Lončarević, Cath Jackson

**Affiliations:** a Institute of Ethnography SASA, Belgrade, Serbia; b World Health Organization Regional Office for Europe, Copenhagen, OE, Denmark; c Institute of Epidemiology, Visegradska 26a, Faculty of Medicine, University of Belgrade, Belgrade, Serbia; d Institute of Public Health of Serbia “Dr Milan Jovanović Batut”, Belgrade, Serbia; e Valid Research Limited, Wetherby, West Yorkshire, UK

**Keywords:** Tailoring immunization programmes (TIP), health communication, vaccination, vaccine hesitancy, health workers

## Abstract

Vaccine communication between health workers and parents affects parental acceptance, so understanding this is particularly important when vaccination rates drop. This paper presents the findings of a qualitative research study conducted in Serbia as part of a Tailoring Immunization Programmes (TIP) project. The aims were to explore the process of vaccination communication between health workers and parents (accepting, indecisive, delaying, refusing), and identify barriers and drivers to effective communication. In-depth interviews with 14 health workers were supplemented and qualified by observations of 40 consultations, using thematic analysis. Study sites were two community health centers in two Belgrade municipalities where a significant drop in childhood vaccination rates had occurred. Key findings were: (1) communication mainly took place between pediatricians and parents, while nurses focused on administering vaccines. (2) Health workers were confident in their skills to communicate and address concerns of accepting and indecisive parents, successfully applying specific strategies. (3) When interacting with delaying and refusing parents, they sometimes agreed to delay vaccination to maintain relationships, confident that most parents would vaccinate in due course. (4) Some refusing parents asked questions grounded in a socio-political agenda regarding vaccines or vaccination. Such questions exceeded the domain of health workers’ expertise, which affected the communication between them. (5) Health workers’ behavior in consultations was sometimes affected by parents’ (dis) trust in their recommendations about vaccination. The study revealed that health workers in Serbia require additional skills and techniques to respond to parents who refuse and wish to delay vaccination, to secure timely vaccination.

## Introduction

Understanding interpersonal communication processes has been recognized as pertinent at a time when many threats to global public health are rooted in human behavior.^1^ For childhood vaccination, the health worker (HW) is often the preferred and most trusted source of information.^2^ Parental desires and expectations related to their interaction with HWs are considerable.^3^ Indeed, some studies suggest that vaccine acceptance is partly determined by physician communication skills.^4,5^ Drawing on research insights into vaccine communication practices between HWs and different groups of parents,^6,7^ researchers have developed suggestions to help HWs better communicate vaccine risks and benefits^8^ and address vaccine refusal.^9^

Serbia was one of the first European countries to introduce vaccines for prevention of communicable diseases, with a mandatory national immunization strategy since 1839.^10,11^ So, there is a long tradition of immunization and many decades of efforts have led to the eradication of poliomyelitis, elimination of diphtheria; whilst other diseases that can be prevented by immunization have been reduced to individual cases.^12^ The childhood immunization programme is delivered free of charge. For almost two centuries, immunization coverage in Serbia has been above 95% for all types of vaccines. However, in recent years coverage for childhood vaccination became suboptimal and fluctuating. This was most significant for the measles-mumps-rubella (MMR) vaccination. Until 2011, MMR coverage in Serbia was above 95%, which is the target for eliminating measles and rubella in the European Vaccine Action Plan 2015–2020.^13^ In 2011, coverage for the first dose administered at 12 months was 98% and 96.5% for the second dose administered to 7-year olds. Then in 2013 it started to decrease and in 2014 it fell to 85.2% and 89.2%, respectively. Further decline in MMR vaccination uptake by 2017 resulted in coverage of slightly more than 60% in the two largest Serbian cities (Belgrade and Nis) with almost half of children eligible for this vaccine, needing to be immunized.^14^ Delays in vaccination as compared with the national recommendations are also a considerable challenge, particularly for the MMR vaccine.^14^

A measles outbreak with almost 6000 cases and 15 deaths was reported from October 2017 until August 2019.^15^ Serbian health authorities responded to the low rates of MMR vaccination by tightening the legislation but failed to sufficiently solve the problem through engaging in active communication with parents and the general public. To address this, Serbia embarked on a project drawing on the World Health Organization (WHO) Tailoring Immunization Programmes (TIP) approach.^16,17^ Using social sciences, ethnographic research techniques and behavioral insights methodology, TIP offers countries a process through which to identify susceptible groups; diagnose barriers and drivers to positive vaccination behaviors and segment populations according to behavioral determinants; in order to design tailored interventions to increase vaccination coverage. To date, TIP projects in the WHO European Region have targeted primary care health workers (Federation of Bosnia and Herzegovina, Bosnia and Herzegovina,^18^ Germany) and medical specialists who advise on vaccination (Armenia); as well specific communities of parents such as the Roma community (Bulgaria), hesitant urban parents (Estonia), internal migrants (Kyrgyzstan), disadvantaged minority communities (Romania), undocumented migrants,^19^ anthroposophic^20^ and Somali communities (Sweden) and a Jewish Charedi community (United Kingdom).^21^

Based on national vaccination coverage data analysis,^14^ existing research^22^ and input from a stakeholder workshop, it was decided to focus the Serbia TIP project on the interaction between parents and HWs with a view to identifying the support HWs may need to facilitate effective vaccination communication. Three inter-linked programmes of work were developed: (1) information for parents, (2) vaccine-related and communication skills training of pediatricians and nurses, and (3) continuing medical education on vaccination and vaccinology.

In this paper we present the first step of programme 2, a qualitative study with HWs (pediatricians and nurses) focusing on their skills and behaviors in communicating with parents of various positions to vaccination. The aims were to:

1. Explore the process of vaccination communication between HWs and parents
2. Identify the barriers and drivers to effective vaccination communication between HWs and parents
3. Explore if, and how, HW vaccination communication varies for parents with different positions to vaccination (accepting, indecisive, delaying, refusing)

## Methods

The research employed two methodological approaches: first in-depth interviews with pediatricians and nurses, followed by observations of pediatricians and nurses in their vaccination consultations with parents (conducted by VT). Whilst parents can discuss vaccination with their child’s pediatrician or nurse during any consultation, these conversations commonly occur during the vaccination appointment. The interviews sought to explore HWs’ experiences of communicating about vaccination with parents, and their views on the barriers and drivers to effective communication. We used observations to supplement and qualify interview findings, basing them on theoretical insights that non-verbal communication, like body language, facial expression or tone of voice, significantly complement verbal communication elements in face-to-face interactions.^23^

### Ethics approval and consent to participate

Ethical approval was secured from the Ethics Committee of the Institute for Public Health of Serbia “Dr Milan Jovanović Batut”. Interview participants received a participant information sheet and agreed to participate and be audio recorded by signing a consent form before the interview commenced. HWs granted verbal consent to be observed during their work. All parents consented verbally to observations upon being verbally informed about the research.

### Setting

Interviews and observations were carried out in two Community Health Centers (CHC) in Belgrade, the capital of Serbia (labeled as CHC 1 and 2). CHC is a primary health care facility that usually covers the territory of one municipality or town. Belgrade is divided into 17 municipalities, each with one CHC. In this study, the municipalities were selected as areas where a significant drop in childhood vaccination rates had been detected in previous years.

### Participants and recruitment

Fourteen in-depth, face-to-face, individual interviews were conducted from June until August 2018 – nine with pediatricians and five with nurses. The heads of pediatric wards in both CHCs circulated study information to HWs and were asked to invite a mix of HWs based on their presumed different approaches in implementing vaccination and communication with parents. All the contacted HWs agreed to participate. The interviews took place in the CHCs, usually before or after a participant’s shift. Due to the large size of the area covered by CHC 1, the interviews were conducted by VT at four different sites with six pediatricians and four nurses. In the smaller CHC 2, three pediatricians and one nurse were interviewed at one site.

Participants in the observations also participated in interviews and were a convenience sample available on the day that the researcher (VT) attended the facility. Three teams (each consisting of one pediatrician and one nurse) were observed during their eight-hour work shift. Two shifts were observed in CHC 1 and one shift was observed in CHC 2. In total, 40 vaccination consultations were observed, each lasting approximately 15 minutes.

All participants were women with extensive work experience. This gender imbalance reflects HWs in Serbia where the vast majority (almost 90%) of both pediatricians and nurses are women.^24^ The pediatricians had over 30 years of work experience, with the exception of one pediatrician with 15 years of experience. The work experience of nurses ranged from 19 to 36 years. This also reflects the age structure of pediatric HWs in Serbia where the average age of pediatricians is 52^24^ and approximately 30% of pediatricians are older than 55 years of age.^25^

### Data collection

The interviews were conducted using a topic guide that was pilot tested with one pediatrician and one nurse. It particularly focused on HWs’ experiences, practices and behaviors related to questions and concerns that parents raised in vaccination consultations. The interviews were audio recorded and lasted between one hour and one hour and a half.

Field notes were made during the observations using a template with pre-determined topics, including consultation time; attending parents; parents’ verbal and non-verbal communication; HWs’ interaction with child; and HWs’ interaction with parents. An additional tool developed within the Sharing Knowledge of Immunization (SKAI) approach^26^ was used to document parents’ questions and HWs’ approach to communicating with parents, e.g., initiation and guiding of conversations.

### Data analysis

Interview recordings were transcribed verbatim and half were purposively selected for translation into English, to include a mix of pediatricians and nurses, across both CHCs. A coding framework was developed (by CJ) based on the topic guide. It was piloted and refined using the seven English transcripts (by VT, CJ). VT then coded the data manually using the Serbian transcripts. The next step was to identify commonly recurring themes across data (by VT, CJ). The approach taken in the analysis sought to reveal participants’ experience and the ways the broader social context impinged on that experience. As observations were primarily meant to supplement interview findings, the analysis was conducted to gain insight into the “actual” behavior of participants in communication and to determine whether observations confirmed or contradicted HWs’ accounts.

## Findings

We present here data on HWs’ approach to vaccination communication depending on the questions and concerns raised by different groups of parents (accepting, indecisive, delaying, refusing). The four parent groups were identified on the basis of HWs’ responses in interviews about their experience with parents. Other studies have classified parents in similar groups according to their attitudes about vaccination.^2,26^

### Communication with parents

The observations revealed that the main communication took place between pediatricians and parents. Nurses were mostly in charge of administering vaccines and providing basic information related to side effects and how to respond to these. This was in line with nurses’ interview statements that pediatricians should be the primary source of information for parents. Pediatricians assumed an active role in consultations by initiating and guiding conversations, typically adopting an educational communication style by giving comprehensive instructions on various health aspects. In the interviews, it was the general perception among pediatricians that some of their colleagues also had an authoritative approach when communicating with parents. The observations revealed that pediatricians usually provided information on which vaccines were due and their possible side effects with fever and swelling at the application site as the most common topics of conversation. Communication to a large degree depended on parents’ interest meaning that pediatricians would give additional information on vaccines only if asked. This slightly contradicted pediatricians’ accounts in interviews where they said that they routinely provided detailed information on vaccines (e.g., about the significance of vaccines for the child and community, or about the timing of vaccines). General communication with parents was observed as relaxed with HWs acting toward children in a warm and friendly way. This supported HWs’ statements about their positive interaction with most parents.

Most parents in the observed consultations accepted vaccination for their child during the consultation (38/40 children); no cases of outright vaccine refusal were observed; two parents delayed vaccination.

### Accepting parents

This category is for parents who agree to have their children vaccinated without hesitation, doubts or distrust toward the HW, vaccines or health authorities. The interviewed HWs described communication with accepting parents as highly satisfactory, often occurring without additional questions.

I start talking, tell them what the child is about to receive, and they say, “No, doctor, we trust you. Apply whatever you say is necessary.”

Pediatrician 2, CHC 1

As indicated above, most parents who were observed in interaction with HWs belonged to this group. This reflects the pattern observed in the national vaccination uptake where the vast majority vaccinate.^14^ Indeed, most of the observed parents did not ask any questions nor raise any particular worries. They also mostly relied on their pediatrician’s decision about vaccination and were ready to take their advice, thereby suggesting trust. When accepting parents asked questions, these referred to strictly medical issues: what illness a vaccine would protect against; which reactions could be expected and what to do should they occur (particularly fever); as well as scheduling of vaccinations. What was noticeable about these inquiries was that they were within HWs’ expertise, who appeared confident in providing answers.

### Indecisive parents

The findings for this parent group were based completely on interview data, as consultations with indecisive parents were not observed. According to the HWs’ experience, these parents could be defined as those who have doubts whether to agree to vaccination or not, and whose indecisiveness is based more on rumors than on having previously developed a clear position on vaccines. The participants believed that these parents tended to resolve their dilemmas by consulting HWs. As the rumors on vaccine safety were the main cause of indecisiveness for these parents, they usually didn’t ask precise questions about vaccines, but mostly tried to establish whether there was some truth to those rumors.

So, they mostly say: “That story, everything that is being said and written, for this reason I have to ask you about it”, that’s usually how it goes. I mean, their questions are: “I would like to hear your opinion. It is important for me to hear what you think about it.” Everything is like, “I have heard this and watched that [on TV or the Internet].” And then when you ask them a concrete question: “And what exactly are the arguments, what does it mean that you have heard something somewhere and who is the one who came up with that story?”, they don’t have a concrete answer. “But where there’s smoke, there’s fire.” I mean, it mostly comes down to that, they have nothing else to tell you.

Nurse 1, CHC 1

Some say: “I have doubts whether to receive that vaccine or not. You see all what is happening.”

Pediatrician 6, CHC 1

HWs’ answers suggested that an important characteristic of indecisive parents was that despite the fear they openly showed, they maintained confidence in pediatricians and nurses, and relied on them to eliminate that fear. The answers suggested that such behavior of indecisive parents resulted in HWs responding with empathy, understanding and encouragement.

I tell them that I understand them [their concerns], but that there is no reason to worry.

Nurse 2, CHC 1

According to HWs’ accounts, the trusting relationship between indecisive parents and HWs brought about specific practices in their communication. One of those was using social instead of strictly medical arguments in favor of vaccination. For example, stressing the common parental identity that HWs and parents shared.

Then, they often ask me: “Did you give that to your child?” I say: “Yes, I did.” That’s the best argument. That has a positive effect. They say, “Ok then.”

Pediatrician 2, CHC 2

Well, a mother used to come upstairs. At the end of our conversation, she told me “If you say so, you who have been doing all those things, and you have vaccinated your own children. Then why do I have the problem with that?” She really said ”for me, those are the strongest arguments I have ever heard. If you have told me so, then there are no more dilemmas for me, I am really going to vaccinate my child now.”

Nurse 1, CHC 1

Another practice the pediatricians stated they used to help these parents make a positive decision on vaccination was providing concrete evidence of tested and confirmed quality and safety of vaccines, addressing widely spread rumors about the quality of vaccines used in Serbia not being as good as in some developed countries.^27^

For instance, we tell them this same lot of vaccine was applied to a child that lived in France. Then I show them that lot number in the records and I say, “Here, see, the French give that same vaccine to their children as we give to ours.” In Germany a child was vaccinated against hepatitis with the vaccine from the same lot that we are using. If they say the vaccine is not good, we tell them, that there is an approval for use under a specific serial number. So they can check everything. Parents check all those things now. The information is available to them on the Internet and they really check. Yes, yes, that helps them.

Pediatrician 3, CHC 1

In HWs’ experience, all these practices had a positive effect on the acceptance of vaccination. Overall, it seemed that these HWs were confident in their skills to communicate and address concerns of indecisive parents.

### Delaying parents

Two parents who were observed in interaction with HWs belonged to this group and both delayed MMR vaccination of their child. One had concerns about their child’s development, and other had decided not to accept any vaccines until the child was nine months of age (we do not know if they received the vaccinations given at birth). Neither seemed to have medically correct contraindications, at the time of the investigation. The pediatrician did not report these cases as refusal.

Unlike indecisive parents who mostly did not show an established clear position on vaccines and vaccination, delaying parents usually had an already formed opinion that children received vaccines too early and that their body was not mature enough for vaccination. They did not reject vaccination, but they also did not want to adhere to the existing schedule. According to the experience of HWs, most of these parents delayed the MMR vaccine, only agreeing to it when they believed that their child was ready, trusting primarily their own judgment. Also, parents whose goal was for their child to receive a certain vaccine as late as possible did not ask questions to explore the issue or learn from the HW.

All of the HWs mentioned that in order to maintain a trustful relationship with this group of parents, they sometimes agreed to postpone vaccination whilst continuing to discuss it with parents, confident in the belief that they will agree to vaccination at some point.

We gave in under their [parents’] pressure, and we waited until (s)he is 15 months old, some of them [parents] wait for two years for no good reason. I don’t know why they delay, but in principle they receive the vaccine. We stretch the timelines a bit, but at least we get them vaccinated.

Pediatrician 5, CHC 1

Pressuring parents by insisting on vaccination “here and now”, was not considered a good approach by any of the participants, because they found the delaying resulted in success for most cases.

Parents usually don’t complain if the physician says, “Vaccinate your child, but fine, you don’t have to do it today.” But physicians who do things strictly by the book, so to say, they have a lot of problems [conflict] with parents.

Pediatrician 1, CHC 2

This discussion was considered to be very demanding work, but often with good results as most parents agreed to vaccination in the end.

“But, think a bit. Do you know how many children have died so far? Do you know how severe was the illness of those children? If you don’t know, do you want me to tell you? And how you die from those illnesses and what it means to develop pneumonia because of measles. Think a bit about those things and then come. See me in two weeks.” And at the same time, you inform them about the legal regulations should they refuse.

Pediatrician 6, CHC 1

HWs described a sub-group of delaying parents who wanted to delay vaccination for longer than the time which the pediatricians were prepared to postpone. Pediatricians expressed their frustration with such parents, especially in cases when they turned to manipulation to delay vaccination for as long as possible, e.g., stating that the child was ill as the reason for not appearing for the scheduled appointment.

“Why did you miss this revaccination, what happened?” Then they say, “He was ill, we travelled.” “But you’re a year late!” Ah, I am rigorous in such cases, those are the patients that you see all the time, you know whether the child was ill or not and whether a parent is avoiding vaccine or not.

Pediatrician 4, CHC 1

Pediatricians’ answers suggested that they had difficulties in reaching agreement on the time of vaccination with such parents; however, they would go to great lengths to avoid reporting parents as refusers.

### Refusing parents

The findings for this parent group are based entirely on data provided in interviews. Parents refusing vaccination can be divided into two subgroups: those who delay vaccination for so long that they shift to the category of refusing parents; and those who make it clear from the outset that they do not want vaccination. Knowing the difference between these groups is important because their different behavior affects the interaction with HWs. In the first case, the procedure is the same as with delaying parents, but with a negative outcome i.e., the child is never vaccinated.

According to HWs’ experience, refusing parents mostly asked the following questions: who could guarantee the vaccine would not harm the child; why were vaccines imported and not produced by the national manufacturer; why was vaccination in Serbia mandatory; what did vaccines contain. HWs’ accounts suggested that such questions would usually turn the communication into discussions departing from strictly medical aspects of vaccines – on vaccination being mandatory, causes for the vaccines not to be produced locally, etc. In HWs’ views, refusing parents had already formed distrust and negative attitudes about vaccines as well as the established vaccination system, and raised these questions in consultations only to contradict HWs as the representatives of that system. As a consequence, most participants stated that they simply could not reach refusing parents.

They have their own sources of information. They do not even ask about our sources of information. When they ask a question, they ask it with the goal to undermine your response. I call that undermining questions. They want to compromise vaccine or to provoke you.

Pediatrician 4, CHC 1

The ones you can’t talk to are those who completely reject vaccination. They stand their ground and nothing helps there, not sending summons, not us convincing them, nothing.

Nurse 3, CHC 1

An additional frustrating aspect identified by pediatricians in communication with refusing parents was their attitude and behavior on such occasions. According to HWs, these parents could sometimes be quarrelsome and hostile. In such cases some pediatricians would not engage in further interaction, while others would get involved in the vicious circle of polemics and attempts to convince them without a positive outcome.

You know what, they simply bully you. Imagine talking to a woman who, first of all, does not have anything to do with medicine and she is badgering me. I end up looking as someone who puts children in enormous danger by giving them medications. And then they accuse us how. Those are horrible words. “How do you even treat children without performing tests? You prescribe medicines without testing them. How do you give injections?” I say, “A child is not allergic, until the allergy is proven.” Without any … no haste, no raised voice, but they eat your soul.

Pediatrician 2, CHC 2

These findings suggested that refusing parents were particularly challenging for HWs to communicate with and reach agreement on vaccination. The behavior of these parents provoked frustration and stress in HWs, as their skills and strategies mostly proved ineffective in implementing vaccination within this group.

## Discussion

This is the first in-depth study in Serbia to explore HWs’ vaccination communication processes with parents, combining interview and observation methodologies. The study provides new, detailed insight on the communication strategies that HWs use, and the challenges they face, particularly in discussing vaccination with parents who delay and refuse childhood vaccination. It builds on a previous study in Serbia in which HW-parent communication was one component of a wider study^22^ and contributes to the small, but emerging evidence base on HW vaccination behaviors in Central and Eastern Europe.^18,28^ More widely it adds to the portfolio of TIP projects^18-21^ and to the body of literature to understand and improve HW vaccination communication behaviors.^2–9^

Several key themes emerged to elucidate the HW-parent vaccination communication process.

First, the theme of trust appeared especially important for understanding HWs’ reactions in communication with different types of parents. HWs were highly satisfied regarding their communication with accepting parents who demonstrated a high degree of trust in their decision about vaccination. Although indecisive parents were nervous about vaccination, they also trusted HWs who in turn usually responded with empathy and understanding. This suggests that parents’ trust was not only important in making a positive decision about vaccination, as reported elsewhere,^29^ but that it can also influence the behavior of the HWs. Conversely, HWs sometimes expressed frustration in communication with parents who, trusting more their own judgment, continued to delay vaccination. Refusing parents were prone to openly demonstrate distrust toward HWs and the vaccination system, adding to more frustration of HWs. Doctors elsewhere also report the challenge of communicating with parents who decline vaccination for their children^18,30^ and feel responsible for ensuring parents vaccinate their children.^31^ Our findings suggested that HWs needed additional skills for conversation with delaying and refusing parents and for reducing the influence of their distrust and behavior to the success of communication.

An important second theme identified for interactions with refusing parents was that they tended to direct the conversation toward social and political issues by asking questions of non-medical nature. Thus, refusing parents raised questions about institutions that guaranteed vaccine quality and safety; vaccination being mandatory; and vaccines not being produced by the national manufacturer. Such questions appeared to exceed the domain of medical expertise and HWs lost the safe ground where they could talk about vaccines as professionals. Scholars usually call for strengthening of vaccine education among HWs^2,32,33^ and advise on using evidence-based data to address parents’ concerns.^34^ Our findings suggested that communication with parents on vaccines and vaccination was not always limited to medical topics. Whilst better information provision may improve vaccination attitudes, this is unlikely to be sufficient in cases where the institutions are mistrusted.^35^ Moreover, it is clear that public health issues cannot be preserved by legislative measures only, if the general population has no trust in healthcare policies.

Another theme that the analysis revealed was accepting delaying of vaccination as a strategy in keeping the door open for implementing vaccination in the end and in keeping good relations with parents. Developing trusting and positive relationships between HWs and parents has been recognized as pivotal for decision-making about vaccination.^29^ Our findings suggested that HWs need support in establishing other strategies to ensure good relations with parents. Delaying was problematic because it involved investing considerable time and energy in following up and persuading parents to vaccinate. Also, the risk of this approach, if used too often, may be that delaying vaccination becomes socially acceptable among HWs and a practice which could have a spill-over effect on parents who increasingly might view delaying as socially (and medically) acceptable.

Communication has been defined as transactional, interactive action taking place in a social and physical context and influencing the behavior of participants.^36^ The transactional nature of interpersonal communication implies that the individuals involved in it affect and are affected by each other’s contribution.^23^ Therefore, the behavior in vaccination communication is shaped by context, role demands placed on participants and their individual characteristics such as knowledge, emotions, motives or expectations, as well as by cultural factors like shared meanings, ideas, beliefs, values and practices.^23^ The fact that HWs highly valued parents’ trust and compliance (and conversely, found it difficult to accept parental resistance) thus might be better understood by considering the social and historical context. Almost all research participants had over 30 years of working experience, and so many were trained and practiced during times with a more top-down approach in public health with an established authority of health professionals and state regulation practices.^27^ This may clash with the preferences and expectations of modern young families, especially those who value individual empowerment and patient-choice.^37^ If so, the authoritative communication style reported by some interview participants may not be conducive for positive vaccination behaviors among parents, and HWs need support in developing new communication skills and approaches in line with the social and political circumstances.

This valuable insight is now being used to adapt an international vaccination communication programme for HWs^38^ to the Serbian context. The TIP project has also developed parent vaccination information resources and delivered a new continuing medical education curriculum on vaccination and vaccinology to 81 HWs across Serbia in 2019. These are important first steps toward improving vaccination consultations; however, a concerted and continued effort over years is required. Alongside TIP, the national immunization programme has intensified efforts to engage with HWs through the network of public health institutes; and with parents via round table events held at CHCs as well as enhanced vaccination communication in the media. More broadly, the insights gained have contributed to a more nuanced understanding of vaccine acceptance, demand and hesitancy in Serbia which can inform the current roll-out of new COVID-19 vaccines.

### Strengths and limitations

The key strength of this study is the use of two complementary qualitative methods in understanding communication between HWs and parents. The combined approach using the same participants enabled us to identify confirming and contradicting aspects of HWs’ accounts. Two templates added value to the observations, one enabling general description and the other^26^ contributing to identifying specific aspects of communication.

It is important to reflect on the limitations of this study. First, the issue of generalizability (as a qualitative concept).^39^ This was a small study, conducted in two CHCs Belgrade, with highly experienced health workers who were invited by their Director to participate. It was summertime where there are less consultations, and at the height of a measles outbreak. It is of course, possible that we may have heard different accounts and observed different vaccination practices at a different time, and in other CHCs in Serbia with younger, less experienced HWs. However, we achieved data saturation (where no new themes were emerging from the interviews) and captured good diversity of views and practices, providing a valuable breadth of insight that indicated both strengths of, and challenges for HWs. This, and the rigor of the study design and conduct, give us confidence in our findings. We did not collect any demographic data from the parents and so cannot comment on how age, education etc. may be associated with their vaccination position. We also acknowledge that no observations were done with indecisive and refusing parents, meaning we were unable to confirm HW accounts about their strategies with these groups of parents. Finally, the researcher’s presence during consultations may have altered HWs’ or parents’ behavior. Future research could usefully include CHCs outside of Belgrade, a broader mix of HWs and of parents’ vaccination positions.

## Conclusions

This qualitative study focusing on HW perspectives provided important insights into the interaction between HWs and parents with various positions on vaccination. It revealed that HWs in Serbia are in need of additional skills and strategic approaches to respond to parents who refuse and wish to delay vaccination, in order to secure timely vaccination. These insights will now be used to inform actions to improve vaccine acceptance and demand in Serbia.
